# To be… an author

**DOI:** 10.1308/rcsann.2025.0096

**Published:** 2025-11

**Authors:** B Rogers

**Figure:**
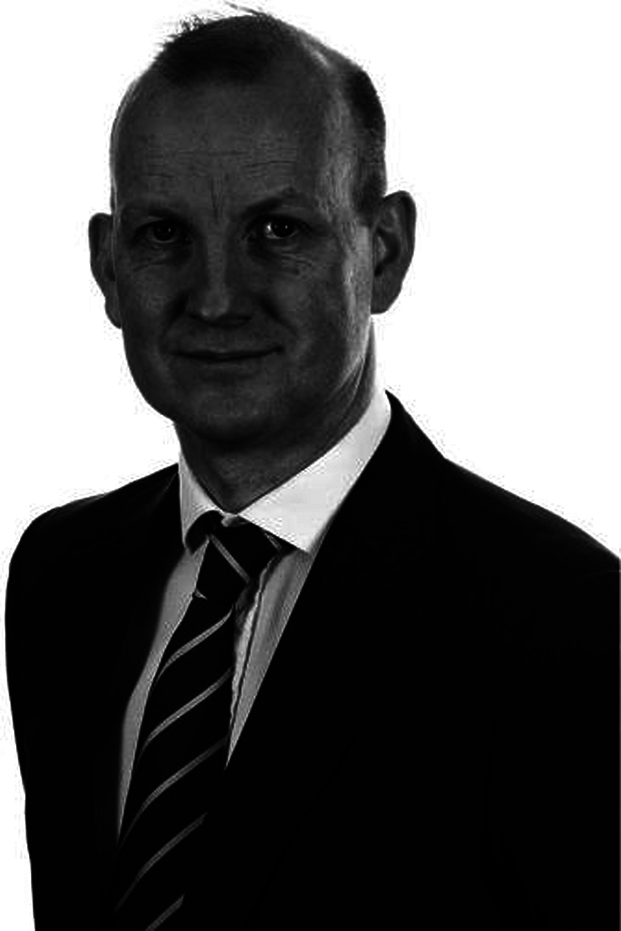


An author of any scientific publication requires numerous attributes, but arguably the most important are responsibility and accountability. This is becoming increasingly important given the changes in the conduct, complexity, and reporting of research.

Artificial Intelligence (AI) has rapidly become a transforming force across numerous sectors, with scientific publishing standing out as an area undergoing significant change. The integration of AI-driven tools and processes is reshaping how research is conducted, reviewed, disseminated, and consumed. There are numerous facets upon which AI may have an on scientific publishing, both positive and negative.

AI-powered applications are increasingly assisting researchers in the preparation of manuscripts. Tools leveraging natural language processing (NLP) can suggest improvements in grammar, clarity, and structure, making articles more readable and professionally presented.

The peer review process, AI is now being deployed to match manuscripts with suitable reviewers based on expertise, previous publication history, and even conflict of interest analysis. It is feasible to utilise machine learning algorithms to flag potential ethical issues, such as plagiarism or data fabrication, before papers reach human reviewers.

Despite its benefits, the adoption of AI in scientific publishing is not without significant concerns. The scholarly publishing community has quickly reported concerns about potential misuse of these language models in scientific publication.^1,2^ Concerns persist around algorithmic bias, transparency, and the potential for misuse, such as the generation of fraudulent papers.

To address concerns about the use of AI and language models in the writing of manuscripts, the Royal College of Surgeons of England (RCS Eng) has updated the journals’ instructions for authors to include guidance on AI and authorship, AI and manuscript preparation and AI and peer review. RCS England journals follow the International Committee of Medical Journal Editors (ICMJE) definition of authors and contributors, and the AI policy is in line with the COPE position statement on Authorship and AI. Readers can find our detailed AI policy here.

The impact of AI on scientific publishing is changing, profound and far-reaching. While AI offers solutions to longstanding problems such as inefficiency and accessibility, it also introduces risks and threats to scientific enterprise.

Undoubtedly, AI will be further and faster developed, being used in all stages of research and the dissemination of information. Guidance for academic publishing, such as that outlined above, will correspondingly need to evolve in time.

However, it is important to protect the integrity and credibility of surgical research, and the trust in medical knowledge.

We unfortunately live in an era of pervasive misinformation and mistrust. To be… an author, the accountability and responsibility for the research presented must remain transparent.

## References

[1] De Waard A. Guest post–AI and scholarly publishing: a view from three experts. *Scholarly Kitchen*. https://scholarlykitchen.sspnet.org/2023/01/18/guest-post-ai-and-scholarly-publishing-a-view-from-three-experts/ (cited October 2025).

[2] Carpenter TA. Thoughts on AI’s impact on scholarly communications? an interview with ChatGPT. *Scholarly Kitchen*. https://scholarlykitchen.sspnet.org/2023/01/11/chatgpt-thoughts-on-ais-impact-on-scholarly-communications/ (cited October 2025).

